# Spatial lipidomic profiling of cutaneous melanoma using electroporation‐based biopsy reveals subtype‐specific metabolic alterations

**DOI:** 10.1002/btm2.70132

**Published:** 2026-04-16

**Authors:** Edward Vitkin, Din Mann, Noam Castel, Omer Yaakov, Vladimir Kravtsov, Klimentiy Levkov, Avshalom Shalom, Alexander Golberg

**Affiliations:** ^1^ School of Mechanical Engineering, Faculty of Engineering Tel Aviv University Tel Aviv Israel; ^2^ Department of Plastic Surgery Meir Medical Center Kfar Sava Israel; ^3^ Department of Pathology Meir Medical Center Kfar Sava Israel; ^4^ Department of Biomedical Engineering Rutgers University Piscataway New Jersey USA

**Keywords:** electroporation‐based biopsy, lysophosphatidylcholine signatures, melanoma lipidomics, spatial metabolic profiling, triacylglycerol depletion

## Abstract

Cutaneous melanoma is a highly aggressive skin cancer with rising global incidence. Although understanding melanoma metabolism requires spatially resolved molecular information, conventional excisional sampling offers limited access to spatial gradients within tumors. Here, we introduce a handheld electroporation‐based biopsy (e‐biopsy) device, a newly engineered tool that delivers controlled pulsed electric fields to transiently permeabilize cells and extract intracellular molecules directly from tissue. This minimally invasive, probe‐based approach enables rapid, localized molecular harvesting without surgical excision, offering a fundamentally new route for in situ tumor profiling. Using this handheld system, we obtained paired molecular samples from tumor centers and margins in 44 locations across 21 melanoma patients, including superficial spreading melanoma and malignant melanoma in situ. Mass‐spectrometry analysis of e‐biopsy extracts identified 85 lipid species and revealed reproducible spatial metabolic patterns. While the device enabled consistent extraction across regions, tumor centers displayed a reduced presence of select triacylglycerols (TG), ether‐linked lysophosphatidylcholines (LPCs), and phosphatidylcholine (PC) PC(32:1). Certain species, including LPC(16:0e) and TG(15:0_16:1_18:1), were absent or markedly diminished centrally. Subtype comparisons showed limited differences at tumor cores but margin‐specific TG variations. This study demonstrates that the handheld e‐biopsy device provides a novel, minimally invasive platform for spatial metabolic characterization of melanoma.

**Abbreviations**
ANOVA: analysis of variance
PI3K‐AKT: phosphoinositide 3‐kinase (PI3K) and protein kinase B (AKT) signaling axis
ROS: reactive oxygen species
UPLC‐MS‐MS: ultra‐performance liquid chromatography–tandem mass spectrometry
UVA: ultraviolet A

**Translational Impact Statement**

We introduce a handheld electroporation‐based biopsy device as a novel, minimally invasive platform for spatial molecular assessment of melanoma. By delivering controlled electric pulses through a portable probe, the device enables precise extraction of intracellular material without surgical excision. This approach reveals metabolic alterations, such as depletion of select triacylglycerols and lysophosphatidylcholines, previously difficult to evaluate. Its scar‐free, repeatable sampling capability supports real‐time metabolic monitoring, enhances patient‐specific risk stratification, and may significantly improve surgical planning and therapeutic decision‐making in skin cancer.

## 1 INTRODUCTION

Cutaneous melanoma affects over 325,000 people worldwide each year and accounts for more than 57,000 deaths annually, making it one of the most aggressive and lethal forms of skin cancer.^1^, ^2^ Given its rapid progression and high metastatic potential, identifying reliable molecular markers is essential not only within the tumor core but also in the adjacent surgical margins, where early signs of invasion, recurrence, or immune evasion may reside. Such markers could improve diagnostic accuracy, guide surgical decision‐making, and enable personalized monitoring of therapeutic response.

Lipidomic profiling has emerged as a promising molecular approach for melanoma assessment, offering insight into tumor biology and metabolic reprogramming when combined with advanced sampling technologies.^3^, ^4^ Lipid metabolism plays a central role in melanoma progression by modulating cell signaling, membrane fluidity, and tumor–microenvironment interactions.^4^, ^5^ Alterations in lipid droplet accumulation and fatty acid metabolism have been linked to melanoma's capacity to adapt to metabolic stress and resist immune surveillance.^6^ Several studies have identified distinct lipidomic signatures that differentiate melanoma from benign and healthy tissues, supporting the development of lipid‐based biomarkers^3^, ^7^, ^8^.

Analytical tools such as mass spectrometry and lipid fingerprint‐based histology have enabled classification of nevi, primary melanoma, and metastatic lesions according to their lipid profiles.^3^, ^7^, ^9^ Additional molecular diagnostic strategies, such as gene expression profiling and mutation detection, offer complementary data but depend on effective molecular sampling from spatially distinct tumor regions.^10^, ^11^

Electroporation‐based biopsy (e‐biopsy) is a minimally invasive method for molecular sampling that uses short electrical pulses to transiently permeabilize cell membranes, allowing the release of intracellular molecules such as lipids and proteins into a surrounding collection medium.^12^, ^13^, ^14^ Unlike conventional biopsy methods, e‐biopsy enables spatially resolved molecular extraction without excision, minimizing tissue trauma while preserving analyte integrity.^12^

In previous work, we demonstrated that e‐biopsy enables differential proteomic profiling of melanoma brain metastases, distinguishing tumor centers, margins, and surrounding healthy tissue in a murine model.^14^ More recently, we applied e‐biopsy to human skin to reveal distinct proteomic and lipidomic profiles in basal cell carcinoma (BCC), cutaneous squamous cell carcinoma (cSCC), and normal tissue.^15^, ^16^, ^17^, ^18^ These findings highlight e‐biopsy's potential for spatial lipidomic profiling in skin cancers, including melanoma.

In this study, we characterize lipidomic differences between three histological subtypes of human melanoma and their adjacent surgical margins. By mapping lipid alterations across these spatial domains, we aim to uncover diagnostic and prognostic markers that could enhance early detection, stratify clinical risk, and inform treatment monitoring through minimally invasive approaches.

## 2 MATERIALS AND METHODS

### 2.1 Clinical trial

From November 2023 to June 2024, 22 patients underwent surgical excision of skin lesions suspected for melanoma, at Meir Medical Center (Kfar Sava, Israel). All lesions excised were at least 1 cm in diameter. Before placing in paraffine and following histopathological examination, proteins were extracted from the fresh samples using e‐biopsy technique. Later, pathological diagnosis confirmed 21 patients with melanoma, specifically 8 of malignant melanoma in situ (MMIS) and 13 of superficial spreading melanoma (SSMM).

### 2.2 Ethics statement

This study was approved by the Meir Medical Center Institutional Review Board (MMC230‐19). All patients gave written consent for participation and genetic analysis of tissues.

### 2.3 Lipidome extraction with e‐biopsy

**FIGURE 1:**
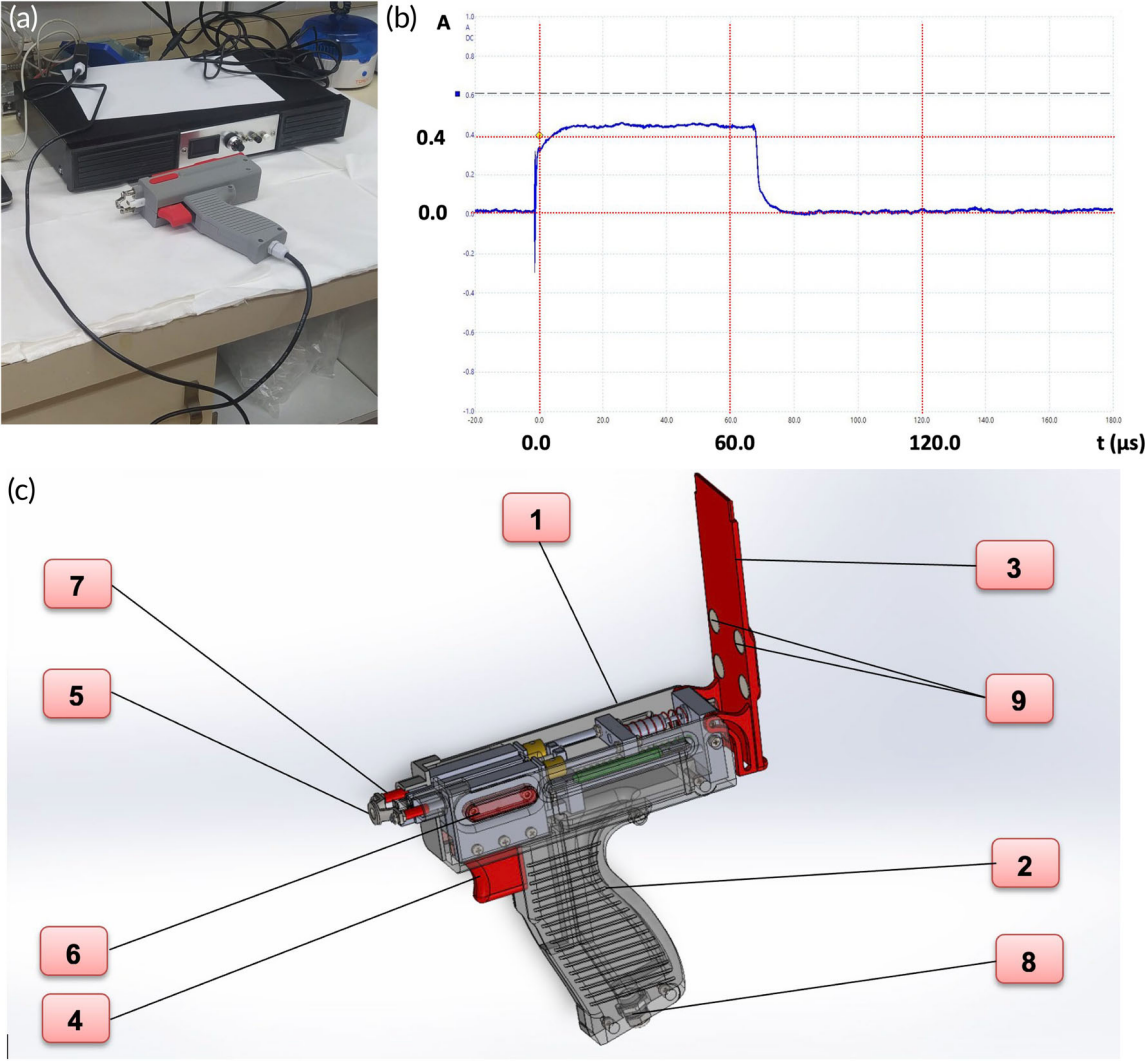
(a) Digital image of the electroporation‐based biopsy devices setup: Custom made battery‐powered pulsed field generator and a handheld probe. (b) Example of the pulse shape as generated by the system and recorded on excised melanoma sample. (c) Detailed design of the E‐probe. (1) Right body part, (2) left body part, (3) syringe lodgment cover, (4) syringe piston driver trigger, (5) annular passive electrode, (6) insulating aps of screw, (7) insulators of rods of passive electrode, (8) high voltage clamp.

Each patient's lesion was sampled in two locations: visible tumor center and excised tissue margin. The sampling was performed using a handheld probe, E‐probe, through which the pulsed electric field is delivered to the tissue and which allows also for sampling the liquids from the electroporated volumes (Figure 1a–c). Additional information about the optimization of E‐probe electrodes design configuration and the details on the battery‐powered pulsed field generator used appears in Ref. [19] Liquid sampling from excised tissue was done using a 30‐G insulin syringe which also served as the positive electrode inserted inside the E‐probe lodgement (Figure 1c). The used pulsed electric field protocol was as follows: applied voltage 65 V, pulse duration 40 μs, number of pulses 500, pulse repetition frequency 4 Hz. Ten seconds after the end of the last pulse the liquids were extracted and transferred to 20 μL double distilled water aliquots. Immediately after the sampling the excised tissue was placed in formaline and sent to standard pathology analysis. The liquids sampled were immediately transferred to 1.5 mL tubes with 100 μL double distilled water and stored at −20°C until shipped to Beijing Genomics Institute for UPLC‐MS–MS analysis.

### 2.4 UPLC‐MS–MS analysis

The UPLC‐MS–MS analysis was performed by Beijing Genomics Institute. An ACQUITY UPLC CSH C18 (1.7 μm, 2.1 × 100 mm, Waters, USA) and Q Exactive mass spectrometer (Thermo Fisher Scientific, USA) were used for lipid analysis. The output of the UPLC‐MS/MS analysis was imported to LipidSearch v.4.1 software (Thermo Fisher Scientific, USA) for molecular identification and quantification. The software was also used to impute missing values. Excel files were generated to include, among other things, the lipid ID, reliability score (graded A to D, with A and B being the most accurately identified lipids used for subsequent differential lipid screening), and the observed intensity of the lipid in the sample (Table S1 and *GitHub*: https://github.com/EdwardVitkin/ebiopsy-melanoma/tree/master/mnscrpt_lipidomics/anlz_lipidomics__no_mixed).

Lipids that appeared in at least 10% of samples in at least 1 comparison group (i.e., in at least two samples in one of SSMM‐Center, SSMM‐Margins, All Melanoma—Center, All Melanoma‐Margins groups OR at least one sample in MMIS‐Center or MMIS‐Margins groups) were defined as eligible for further analysis.

### 2.5 Bioinformatics analysis

**FIGURE 2:**
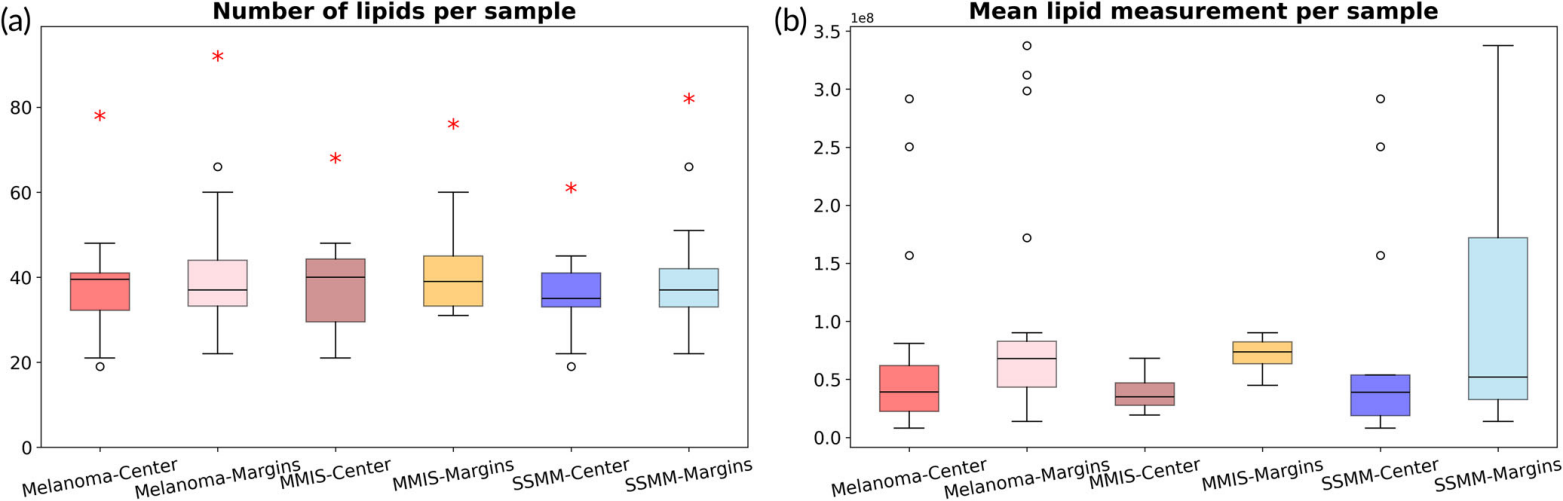
(a) Number of lipids identified in the sample. The red marker displays the number of lipids observed in the entire subset of samples. (b) Mean lipid intensity in the sample. In both figures, the box represents the inter‐quartile range (IQR), its internal line indicates the median, the whiskers extend to 1.5 × IQR, and small circles denote outliers. MMIS, malignant melanoma in situ; SSMM, superficial spreading melanoma.

Per sample visualizations (Figure 2) were performed with the *matplotlib.pyplot.boxplot* function with default parameters. Multi‐group comparisons were performed with the one‐way ANOVA test (*scipy.stats.f_oneway*), while pairwise Center versus Margin comparisons were performed with the dependent Student *t*‐test (*scipy.stats.ttest_rel*).

Pairwise differential expression analyses of lipids between sample groups (i.e., Center vs. Margin) were performed (i) over the change in average (over group) measured lipid intensity and (ii) over the change in lipid appearance patterns between two groups. First was tested with a non‐parametetric Wilcoxon Signed‐rank test (*scipy.stats.wilcoxon*); and the second was tested with a Vitkin proportion‐based test.^20^ The false discovery rate (FDR) was calculated as the ratio between the expected and observed numbers of significant results at a chosen uncorrected *p*‐value threshold (e.g., 0.05). An FDR of 1.0 indicates that the number of observed significant results equals the number expected by random chance, which in this case corresponds to 2.13 observations per tail for the 5% *p*‐value cutoff across 85 lipids.

## 3 RESULTS

### 3.1 Handheld E‐probe development for electroporation‐based biopsy

To enable minimally invasive molecular sampling, we developed a handheld e‐biopsy (E‐probe) device engineered for controlled delivery of pulsed electric fields to targeted tissue regions. In this study, the E‐probe is integrated with a battery‐powered pulse generator^19^ and a compact electrode assembly that simultaneously applies electroporation pulses and collects released intracellular material. The device's ergonomic form factor allows stable one‐hand operation, enabling clinicians to position the probe precisely on irregular skin surfaces. Its internal electrode geometry is optimized to generate uniform electric fields across a confined sampling volume, ensuring reproducible electroporation.^19^ A new feature is a built‐in syringe‐driven collection channel that permits immediate retrieval of extracellular fluid released during electroporation, streamlining the sampling workflow.

### 3.2 Lipidomic high‐throughput measurements of melanoma samples

To investigate the changes in lipidomics patterns between melanoma and its adjusting areas, we performed MS‐based lipidomics on a total of 42 freshly excised tissue samples from 21 patients (two samples per patient), consisting of 21 patients with melanoma, specifically 8 of MMIS and 13 of SSMM (Table 1, Section 2). A pair of samples was collected from each patient—one from the visible center of the lesion (here referred to as Center) and another outside of visible melanoma margins (here referred to as Margins).

**TABLE 1:** Demographics distribution of patients.

Condition	Age	Male	Female	Total
MMIS	64 ± 22	7	1	8
SSMM	74 ± 7	7	6	13

Abbreviations: MMIS, malignant melanoma in situ; SSMM, superficial spreading melanoma.

We employed MS in a data‐independent acquisition method to quantify the lipidome and processed the received MS readings with MaxQuant, resulting in the identification of 99 lipids, out of which 85 were defined as eligible for further analysis (Section 2).

The total number of lipids identified in each subset was comparable, demonstrating consistent and robust lipidomic coverage across all clinical sample types (Figure 2a). While a slight decrease in the number of observed lipids was noted in samples from the center, this reduction was not statistically significant. However, quantitative analysis revealed a significant decrease in lipid levels in MMIS‐Center samples compared to MMIS Margin samples (*p*‐value = 2.0e−03) (Figure 2b). In contrast, the same trend in SSMM samples was not statistically significant (*p*‐value = 0.35), highlighting a distinct difference in lipid distribution and characteristics between the two melanoma subtypes.

### 3.3 Differential expression analysis of lipidomic changes in superficial spreading melanoma and its margins

**FIGURE 3:**
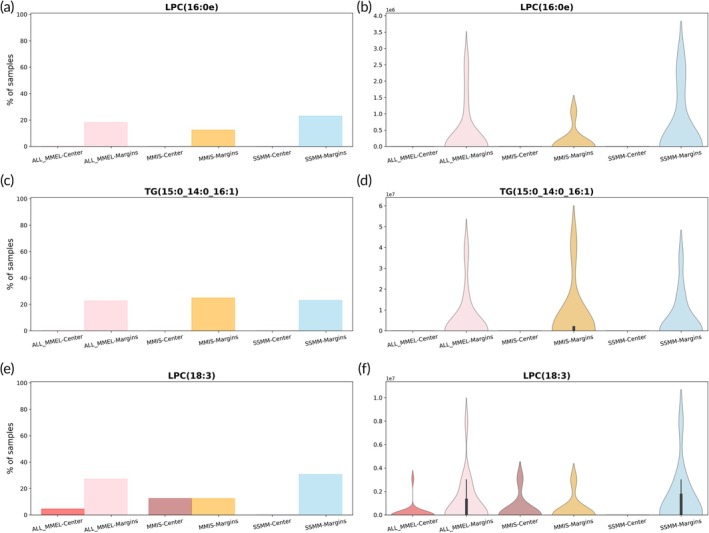
Distribution of selected lipids from Table 2. Bar‐plots (a), (c), (e) show the percentage of samples this lipid was observed. Violin‐plots (b), (d), (f) show distributions of measured lipid intensities over the samples. LPC, lysophosphatidylcholines; MMIS, malignant melanoma in situ; SSMM, superficial spreading melanoma; TG, triacylglycerols.

A lipidomic comparison of e‐biopsy samples collected from the centers of SSMM lesions (Center) and paired samples from outside the visible margins (Margins) across 13 SSMM patients revealed very few statistically significant differences (Section 2). Among the lipids analyzed, only two—LPC(18:3) and TG(15:0_16:1_18:1)— emerged as potentially noteworthy (Table 2, Figure 3).

**TABLE 2:** Melanoma‐Center versus Margins lipidomics comparison. Percentage value for each lipid corresponds to its relative measured intensity in lesion Center versus lesion Margin. 0% means that the lipid was not observed in any Center sample.

Lipid Id	Lipid formula	All melanoma	MMIS	SSMM
LPC(16:0e)^a^	C24 H53 O6 N1 P1	**0.0%**		
LPC(18:1e)	C26 H55 O6 N1 P1	**0.0%**		
TG(15:0_14:0_16:1)^a^	C48 H94 O6 N1	**0.0%**		
LPC(18:3)^a^	C26 H49 O7 N1 P1	15.3%		**0.0%**
PC(32:1)	C40 H79 O8 N1 P1	17.9%		
TG(19:1_18:1_18:1)	C58 H110 O6 N1	45.5%	29.1%	
TG(15:0_18:1_18:1)	C54 H104 O6 N1	58.0%	48.5%	
TG(17:0_18:1_18:1)	C56 H108 O6 N1	58.2%	48.1%	
TG(18:1_17:1_18:1)	C56 H106 O6 N1	58.3%	59.4%	
TG(15:0_16:1_18:1)	C52 H100 O6 N1	60.2%	38.5%	63.3%
TG(15:0_18:1_18:2)	C54 H102 O6 N1	60.4%	54.9%	
TG(16:1_16:1_18:2)	C53 H98 O6 N1	63.8%		
TG(16:0_14:0_16:1)	C49 H96 O6 N1	64.3%	32.1%	
TG(18:1_18:1_18:1)	C57 H108 O6 N1	64.6%	56.6%	
TG(16:0_16:1_16:1)	C51 H98 O6 N1	64.9%	42.7%	
TG(16:0_18:1_18:2)	C55 H104 O6 N1	66.3%	66.8%	
TG(16:0_14:0_18:1)	C51 H100 O6 N1		49.0%	
TG(16:0_16:0_18:1)	C53 H104 O6 N1		51.4%	
TG(16:0_17:0_18:1)	C54 H106 O6 N1		40.4%	
TG(16:0_18:1_18:1)	C55 H106 O6 N1		52.3%	
TG(18:0_16:0_18:1)	C55 H108 O6 N1		49.8%	
TG(18:0_18:0_18:1)	C57 H112 O6 N1		50.1%	
TG(18:0_18:1_18:1)	C57 H110 O6 N1		48.3%	
TG(20:1_18:1_18:1)	C59 H112 O6 N1		48.8%	

Abbreviations: LPC, lysophosphatidylcholines; MMIS, malignant melanoma in situ; PC, phosphatidylcholine; SSMM, superficial spreading melanoma; TG, triacylglycerols.

Note: Bold values indicate 0% relative frequency, denoting absence of lipid in CENTER group.

^a^ Distribution of lipids are displayed in Figure 3.

LPC(18:3) displayed a significant (*p*‐value = 0.030) difference in its appearance pattern. It was observed in 4 (out of 13, which is 30%) of Margin samples, while completely missing from Center samples. TG(15:0_16:1_18:1), which was identified in the majority of samples in both locations (10 times in Center and 12 times in Margins), displayed a statistically significant (*p*‐value = 0.048) but slight (37%) decrease in its measured amount in Center samples (Section 2).

### 3.4 Differential expression analysis of lipidomic changes in malignant melanoma in situ and its margins

The lipidomic comparison of e‐biopsy samples collected from the centers of MMIS lesions (Center) and paired samples from outside the visible margins (Margins) in eight MMIS patients revealed a few notable findings (Table 2).

The measured intensity increased significantly for a single (FDR = 2.13) lipid DG(18:1_18:1). In contrast, 18 lipids (FDR = 0.12) were measured with significantly (*p*‐value <0.05) lower intensity in MMIS‐Center samples compared to MMIS‐Margins samples. For example, one such lipid is TG(19:1_18:1_18:1), whose intensity in Center samples decreased to less than 30% (*p*‐value = 0.02) relative to its intensity in Margins.

### 3.5 Differential expression analysis of lipidomic changes in combined melanoma data and its margins

Differential expression analysis of data from all 21 melanoma patients in centers (here Melanoma‐Center) relative to its paired margins (here Melanoma‐Margins) shows similar results to that observed when analyzing each main subtype individually. No lipid has displayed a statistically significant increase of its levels in Melanoma‐Center, while 16 lipids did show a decrease (Table 2, Figure 3).

We observed a significant decrease in the appearance for seven lipids (FDR = 0.30), out of which TG(15:0_14:0_16:1), LPC(16:0e), and LPC(18:1e) were identified solely in Melanoma‐Margins (23%, 18%, and 18% of samples, respectively). Analysis of changes in intensity levels showed 11 lipids (FDR = 0.19) that on average showed a 40% ± 6% decrease in Melanoma‐Centers compared to their paired Melanoma‐Margins samples (Figure 3). For example, TG(15:0_16:1_18:1) lipid intensity in Melanoma‐Centers is about 60% of its Melanoma‐Margins values (*p*‐value = 4.2e−03).

### 3.6 Differential expression analysis of lipidomic changes in superficial spreading melanoma and malignant melanoma in situ

**FIGURE 4:**
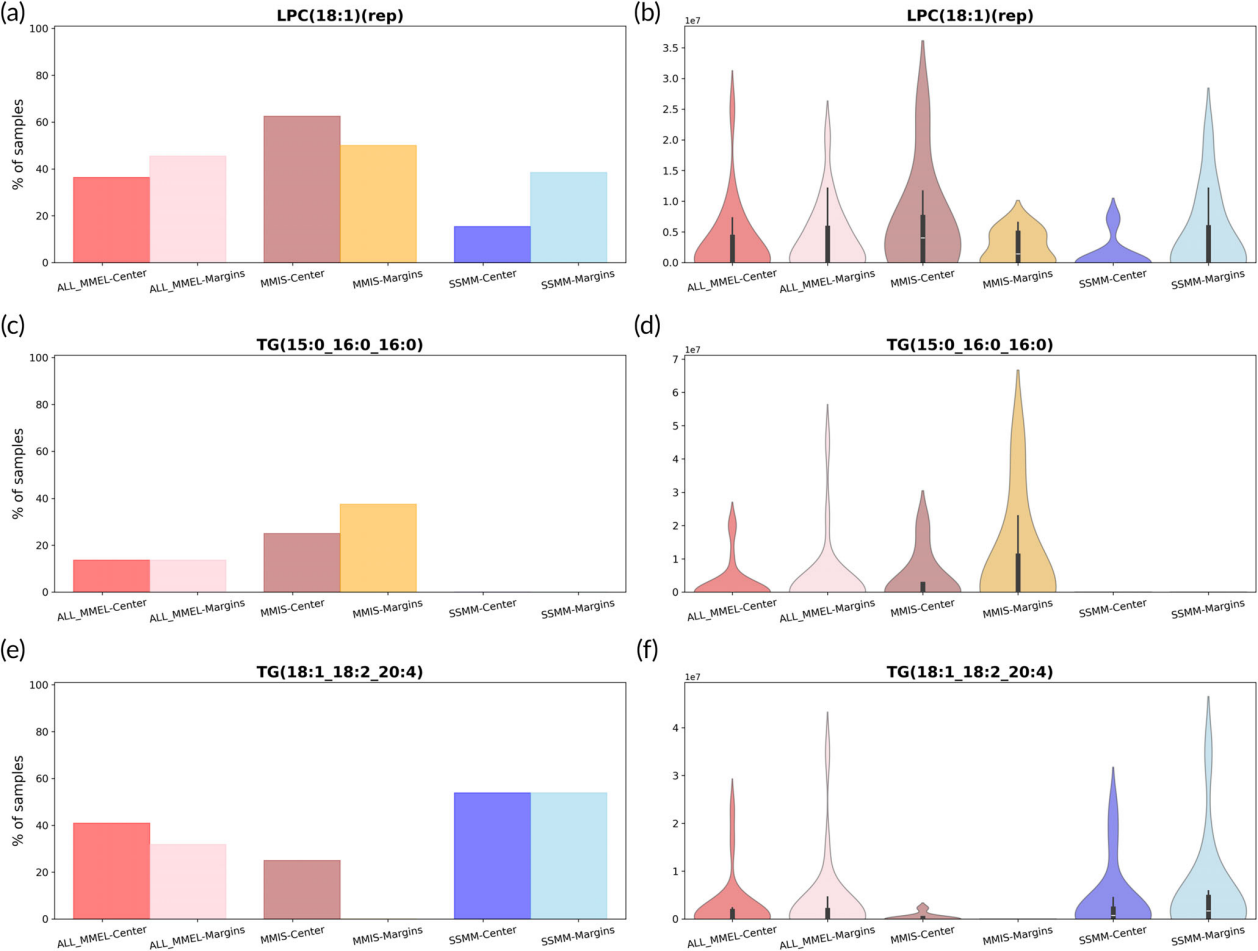
Distribution of selected lipids from Table 3. Bar‐plots (a), (c), (e) show the percentage of samples this lipid was observed. Violin‐plots (b), (d), (f) show distributions of measured lipid intensities over the samples. LPC, lysophosphatidylcholines; MMIS, malignant melanoma in situ; SSMM, superficial spreading melanoma; TG, triacylglycerols.

Comparison of the lipidomics measurements between two melanoma subtypes, SSMM and MMIS, did not result in a significant number of differentially expressed lipids. The only significant change between lesion centers was LPC(18:1)(rep), that was observed in five (62.5%) out of eight MMIS‐Center samples, while only in two (15.4%) of SSMM‐Center samples (*p*‐value = 0.026). The differences between Margins samples were slightly more significant. We identified five triglycerides that were significantly different in MMIS‐Margins samples (two up and three down) relative to SSMM‐Margins (Table 3, Figure 4). The lower number of differences between lesion centers compared to their margins suggests a greater lipidomic similarity between lesion centers of both melanoma subtypes samples compared to their margins.

**TABLE 3:** Differences between malignant melanoma in situ (MMIS) and superficial spreading melanoma (SSMM).

Lipid Id	Lipid formula	Direction (in MMIS)	MMIS‐Center	MMIS‐Margins	SSMM‐Center	SSMM‐Margins
LPC(18:1)(rep)^a^	C26 H53 O7 N1 P1	Center UP Proportion *p* = 0.026 Wilcoxon *p* = 0.089	62.5% (6.34e+06)	50.0% (2.39e+06)	15.4% (1.10e+06)	38.5% (4.21e+06)
TG(15:0_16:0_16:0)^a^	C50 H100 O6 N1	Margins UP Proportion *p* = 0.017 Wilcoxon *p* = 0.158	25.0% (3.77e+06)	37.5% (9.36e+06)	0.0%	0.0%
TG(18:0_16:0_16:0)	C53 H106 O6 N1	Margins UP Proportion *p* = 0.049 Wilcoxon *p* = 0.017	75.0% (3.84e+06)	75.0% (6.70e+06)	53.8% (1.71e+06)	30.8% (1.10e+06)
TG(18:0_18:0_18:1)	C57 H112 O6 N1	Margins UP Proportion *p* = 0.243 Wilcoxon *p* = 0.030	87.5% (5.93e+06)	100.0% (1.18e+07)	76.9% (7.97e+06)	84.6% (7.55e+06)
TG(18:1_18:2_20:4)^a^	C59 H104 O6 N1	Margins DOWN Proportion *p* = 0.011 Wilcoxon *p* = 0.043	25.0% (3.66e+05)	0.0%	53.8% (3.81e+06)	53.8% (5.18e+06)
TG(18:1_18:1_20:3)	C59 H108 O6 N1	Margins DOWN Proportion *p* = 0.154 Wilcoxon *p* = 0.030	50.0% (9.47e+06)	37.5% (4.36e+06)	61.5% (4.11e+07)	69.2% (6.45e+07)

Abbreviations: LPC, lysophosphatidylcholines; TG, triacylglycerols.

^a^ Distribution of lipids are displayed in Figure 4.

## 4 DISCUSSION

This study provides new insights into the lipidomic landscape of cutaneous melanoma by employing e‐biopsy sampling to compare tumor centers and adjacent surgical margins at the molecular level. Using high‐throughput mass spectrometry, we profiled 85 eligible lipid species across 42 paired samples from 21 patients, revealing both subtype‐specific and general trends in lipid alterations. These findings demonstrate the feasibility of e‐biopsy as a minimally invasive molecular sampling method that can capture the spatial dynamics of lipid metabolism in melanoma. On the limitations of this work is an imbalance in the male–female ratio within the MMIS subgroup, which represents a demographic limitation that may influence observed metabolic differences.

Consistent with previous reports highlighting the central role of lipid metabolism in melanoma progression,^9^, ^21^ we observed significant differences in lipid abundance and detection frequency between tumor cores and perilesional tissue. Lipid classes such as triacylglycerols (TGs), lysophosphatidylcholines (LPCs), and phosphatidylcholines (PCs) contributed substantially to this divergence. These classes have been previously implicated in key cancer‐related processes including membrane remodeling, energy storage, immune evasion, and cellular signaling.^7^, ^22^

LPC species, including the ether‐linked LPC(16:0e), LPC(18:3), and LPC(18:1e), depleted at the tumor center versus surgical margins (Table 2), are emerging as functionally significant lipids in melanoma biology.^7^ Although direct studies on LPC(16:0e) are currently limited, several related LPCs—particularly LPC(16:0)—have been associated with early metastatic spread to lymph nodes in melanoma patients.^7^ Lipidomic profiling indicates that while total plasma lipid content tends to decrease during metastasis, specific LPCs are paradoxically elevated and may serve as early biomarkers of disease progression.^7^ Mechanistically, LPCs act as substrates for autotaxin (ATX), an extracellular enzyme that converts LPC to lysophosphatidic acid (LPA), a potent bioactive lipid that promotes tumor cell proliferation, migration, and angiogenesis. Inhibition of the LPC–ATX–LPA axis using LPC‐mimetic compounds has been shown to suppress melanoma progression in preclinical models.^23^ Given the chemical stability and resistance to oxidative degradation of ether‐linked species like LPC(16:0e), their depletion in tumor centers—as observed in this study—may reflect shifts in membrane composition, impaired ether lipid biosynthesis, or altered lipid signaling under tumor‐specific metabolic stress.

PC (32:1) (PC(32:1)), also significantly reduced at the tumor center versus margins (Table 2) a monounsaturated phosphatidylcholine (MUPC), has emerged as a recurrently altered lipid species across multiple cancer types. It has been reported to be significantly elevated in cancerous tissues compared to adjacent normal tissue in breast, lung, colorectal, esophageal, gastric, and thyroid cancers, highlighting its potential as a pan‐cancer metabolic marker.^24^ In the context of melanoma, PC(32:1) is consistently upregulated following UVA exposure, where it contributes to membrane biogenesis and supports tumor cell proliferation under oxidative stress conditions.^25^ Interestingly, treatment with cannabidiol (CBD) and cannabigerol (CBG)—two phytocannabinoids known to induce cellular stress—results in a marked reduction of PC(32:1) levels, suggesting that this lipid may serve as a dynamic and modifiable marker of metabolic adaptation in melanoma.^25^ In our study, PC(32:1) exhibited reduced abundance in tumor centers relative to margins, potentially reflecting impaired phospholipid synthesis, enhanced membrane turnover, or altered remodeling under nutrient or oxidative constraints. These findings align with recent work by Gurung et al.,^26^ who demonstrated that PC, particularly secreted by young subcutaneous adipocytes, is actively taken up by melanoma cells where it stimulates the PI3K‐AKT signaling axis, enhances oxidative phosphorylation (OXPHOS), and increases ROS production. This metabolic reprogramming was shown to suppress metastatic potential and shift tropism toward the lung. The consistent reduction of PC(32:1) in melanoma tumor centers in our cohort may reflect similar spatial constraints on PC uptake or remodeling, supporting its role as a marker of oxidative metabolic stress and spatially restricted tumor proliferation.

TGs, key components of cellular lipid storage, were consistently reduced in melanoma tumor centers compared to adjacent margins in melanoma and its two studies subtypes (Table 2), suggesting a localized disruption in energy storage and lipid buffering. TGs are known to accumulate in lipid droplets and serve as reservoirs for fatty acids under metabolic stress. In several cancers, including melanoma, dysregulated TG metabolism has been linked to altered energy demands, increased β‐oxidation, and enhanced lipolysis to support rapid tumor proliferation and survival under oxidative or nutrient‐limited conditions.^27^, ^28^, ^29^, ^30^ Although specific TG species such as TG(15:0_16:1_18:1) and TG(19:1_18:1_18:1) identified in this study have not been widely reported in melanoma, their significant reduction in tumor cores may reflect increased catabolic activity or impaired lipid droplet formation. Previous work has shown that TG depletion is associated with metabolic reprogramming in melanoma cell lines and reduced capacity to buffer reactive oxygen species through lipid droplet‐mediated sequestration.^9^, ^10^ Gurung et al. reported that melanoma cells exposed to lipid‐rich microenvironments show elevated OXPHOS and ROS levels, while those in aged, lipid‐scarce settings—with lower TG availability—are more prone to visceral (especially liver) metastases.^26^ Our observation that several TG species (e.g., TG(15:0_16:1_18:1), TG(19:1_18:1_18:1)) are depleted in tumor centers is consistent with this metabolic polarization, suggesting localized lipid scarcity and a potential shift toward β‐oxidation‐driven energy production in tumor cores. These spatial TG gradients could be indicative of a metabolically adaptive tumor subregion with altered proliferative and metastatic capacity. Thus, the observed spatial depletion of TGs in melanoma may signal a shift toward oxidative metabolism and a heightened state of metabolic vulnerability. These findings support the role of TGs not only as indicators of metabolic state but also as potential targets for disrupting melanoma cell survival under stress. While our study demonstrates the utility of e‐biopsy for spatially resolved lipidomic analysis, it is limited by cohort size and the inherent heterogeneity of melanoma subtypes. Further studies with expanded cohorts—including nodular and acral lentiginous melanoma—are warranted to validate these findings and identify lipid signatures predictive of clinical outcomes.

Incorporating complementary omics data, such as transcriptomics and proteomics, could provide mechanistic context for lipidomic alterations. Longitudinal sampling during therapy would also enable exploration of lipid‐based markers for therapeutic response or resistance. Given the emerging role of lipid metabolism in immunotherapy outcomes.^31^ integrating immune phenotyping with lipidomics may further enhance melanoma stratification and management. Our study supports lipidomics sampled by e‐biopsy promise for improving melanoma diagnosis, stratification, and treatment monitoring.^32^

## 5 CONCLUSION

This study presents a spatially resolved lipidomic analysis of human cutaneous melanoma using e‐biopsy, revealing distinct molecular differences between tumor centers and adjacent margins. By profiling 85 lipid species across 21 paired samples, we identified consistent reductions in TGs, ether‐linked LPCs, and PCs within tumor cores. These findings suggest that lipid depletion in melanoma centers may reflect increased β‐oxidation, oxidative stress, and impaired lipid droplet formation—hallmarks of metabolic reprogramming in aggressive tumors.

Lipid species such as TG(15:0_16:1_18:1), LPC(18:3), and PC(32:1) emerged as spatially modulated markers, with notably reduced levels in tumor centers compared to surrounding tissue. In MMIS, these reductions were statistically significant, highlighting a potential link between lipidomic remodeling and melanoma subtype or stage. Furthermore, differential lipid profiles in tumor margins between SSMM and MMIS suggest microenvironmental contributions to tumor behavior, which may influence immune response, invasion, and recurrence risk. Our results align with recent studies demonstrating that lipid‐rich stromal environments modulate melanoma metabolism and metastasis. Integration of our clinical e‐biopsy data with such mechanistic insights underscores the diagnostic and therapeutic relevance of lipid species in melanoma. While limited by sample size and histological diversity, this work establishes proof‐of‐concept for using e‐biopsy to interrogate tumor lipid architecture. Future studies incorporating larger cohorts, longitudinal sampling, and complementary omics will help validate lipid signatures for clinical use. Overall, this study advances minimally invasive molecular profiling in oncology and supports lipidomics as a valuable tool for melanoma classification and personalized management.

## AUTHOR CONTRIBUTIONS

**Edward Vitkin**: data analysis, manuscript drafting. **Din Mann**: experimental. **Noam Castel**: experimental. **Omer Yaakov**: manuscript review. **Vladimir Kravtsov**: pathology. **Klimentiy Levkov**: experiments, software, hardware. **Avshalom Shalom**: conceptualization, experiments, data analysis, manuscript drafting. **Alexander Golberg**: conceptualization, experiments, data analysis, manuscript drafting.

## FUNDING INFORMATION

The authors thank the TAU SPARK fund, The Zimin Institute for Engineering Solutions for Advancing Better Lives, and the EuroNanoMed MATISSE project for their support of this project.

## CONFLICT OF INTEREST STATEMENT

Edward Vitkin, Avshalom Shalom, and Alexander Golberg are consultants to Elsy Medical.

## Supporting information


**Table S1.** List of all identified lipids in all samples including their intensity.

## Data Availability

The data that supports the findings of this study are available in Supporting Information S1 of this article and in https://github.com/EdwardVitkin/ebiopsy‐melanoma/tree/master/mnscrpt_lipidomics/anlz_lipidomics__no_mixed.
